# Bioeconomic modeling for a small-scale sea cucumber fishery in Yucatan, Mexico

**DOI:** 10.1371/journal.pone.0190857

**Published:** 2018-01-09

**Authors:** Alvaro Hernández-Flores, Alfonso Cuevas-Jiménez, Alicia Poot-Salazar, Alfonso Condal, Juan Carlos Espinoza-Méndez

**Affiliations:** 1 Universidad Marista de Mérida, Periférico norte tablaje catastral, Carretera Mérida-Progreso, Mérida, Yucatán, México; 2 Centro Regional de Investigaciones Pesqueras de Yucalpetén, Instituto Nacional de Pesca, Boulevard del Pescador S/N, Puerto de Abrigo, Progreso, Yucatán, México; 3 Département des Sciences Géomatiques, Laval University, Quebec, de la Concorde Saint-Romuald, Quebec, Canada; University of Minnesota, UNITED STATES

## Abstract

Due to the heavy exploitation of holothurians over the last few decades, it is necessary to implement fishing regulations aimed at reversing this situation. Holothurians require specific regulations that take into account their biology and ecology. Their behavior to group and form patches as a strategy for feeding, defense and reproduction, makes them vulnerable to overfishing. The higher the population density, the higher the catchability coefficient, and because they are sedentary organisms, the catchability does not change significantly until the density is very low. Hence, the stock assessment of holothurians can be improved by analyzing their spatial distribution. This paper proposes a stock assessment technique that considers the spatial distribution pattern of the sea cucumber *Isostichopus badionotus* from Yucatan, Mexico. A bioeconomic spatial model was developed to explain the interactions between fishing effort allocation, quasi-profits and the population in the short term. Because of the high price of the species and the low production costs, artisanal fishers preferred to maximize short-term quasi-profits, rather than the long-term benefits they could gain with low fishing mortality rates.

## Introduction

The declining trend in abundance of commercially important sea cucumbers worldwide has raised interest in improving their management. Despite the implementation of different strategies to overcome this situation [1–6], progress has been limited. The main factors that explain this situation are the expansion of the Hong Kong market, the development of new fisheries further from Asia, and the use of better fishing technologies [7]. How can small-scale fisheries cope with this situation? Understanding these drivers is important in designing measures to mitigate the impact of fishing pressure; however, it is equally important to understand the functioning of management systems at the local level. Likewise, increasing the knowledge base of the biology and ecology of sea cucumbers, as well as the economics of the fisheries can contribute to a significant improvement in their management [7–16].

Proper fisheries management must be based on reliable stock assessments, which require a comprehensive knowledge of the species. In the case of holothurians, being a sedentary species, their spatial distribution is key for certain biological processes such as social behavior for feeding, defense, and reproduction. Sea cucumbers are gonochoric and iteroparous species [17], with external fertilization [18,19]. Their reproduction depends on a number of factors, but the proximity of spawners and aggregative behavior [20] are very important.

Because many species of sea cucumbers form patches on the seafloor, stock assessment methods should avoid the assumption of uniform distribution. Therefore, population density becomes an important variable for stock assessment and management purposes, since the areas with higher density and abundance may be more susceptible to exploitation. In this way a healthy population of sea cucumbers without adequate regulations could be overexploited quickly, since fishermen would alter their natural spatial structure in a short time. In such a case, a management strategy could dictate limiting fishing mortality based on the knowledge of the average density that guarantees the biological strategies of the species, including reproduction, defense or feeding [8, 21].

In order to assess and portray the performance of fisheries management, bioeconomic models provide a good tool for understanding the functioning of fisheries systems [22]. These models integrate different components (e.g. biological, technological and economic) in one system [23]. Using a dynamic, spatial bioeconomic model, this paper illustrates how the depletion of a sea cucumber fishery can occur in the short term. Different types of information were integrated into the dynamic bioeconomic model, which simulated the population dynamics and the fishing effort allocation. Our results confirmed that sea cucumber fisheries can be overexploited in the short term if appropriate regulations are not implemented. The three-row sea cucumber *Isostichopus badionotus* (Stichopodidae) fishery, from Yucatan was used as a case study.

## Methods

The study area is not a natural protected area, but a portion of Mexico’s territorial sea, and the sea cucumber *I*. *badionotus* is not an endangered or protected species in Mexico. There are numerous fishing villages along the shore of the Yucatan Peninsula, where traditional small-scale fishers target finfish, sharks, octopus, lobster and other mollusks and crustaceans [24]. For decades, fishers ignored the presence of sea cucumbers. In the early 1990s, the *I*. *badionotus* fishery began in Cuba, Panama, Venezuela, Brazil and Bonaire [25, 26, 27, 28]. In 1992 this fishery began in Mexico as an exploratory fishery.

*I*. *badionotus* is widely distributed in the Caribbean [26,27,29,30]. It is found from the shore to a depth of 65 m [31]. Adults can reach up to 45 cm in length. Its development consists of three larval stages: auricularia, doliolaria, and penctactula, which becomes a benthic juvenile. In a laboratory, each female can spawn several times, at least once per month, and has an average fecundity of 200 000 eggs, with a maximum of 1 066 000 eggs [19]. Fertilization is external and after the fertilized eggs hatch into planktonic free-swimming larvae, it takes from 19 to 22 days [19] to settle on places with adequate substrate, food and environmental conditions. Juveniles of *I*. *badionotus* feed on diatoms and detritus of calcareous algae such as *Jania adherens*. Juveniles have cryptic habits and remain hidden under rocks or between coral reefs for about eight months. Upon reaching larger sizes, they move to nearby sands to feed on plant detritus and continue growing until maturity [32]. The size of first sexual maturity is 23.3 cm dorsal length, which is equivalent to an age of 2.5 years. Other reports for Panama and Venezuela mention that the size of first sexual maturity is 15 cm and 18 cm, respectively [27], compared to 22 cm in Cuba.

From an extensive list of holothurians from around the world, *I*. *badionotus* is considered of medium commercial value on the Asian market [29]. However, in 2012 the local price of the species in Yucatan increased so much that hundreds of boats suddenly entered the fishery in a frantic race. As a result of this increased demand, the commercial fishery of *I*. *badionotus* was authorized in Yucatan in 2012. At that time, the lack of scientific knowledge on the species meant that appropriate regulations were not endorsed, resulting in a weak regulatory system. As part of the regulations, only 350 licenses for boats were authorized, with a total allowable catch of 2100 tonnes and a fishing quota per trip of 250 kg (wet weight). In addition, the fishing season was set to two months and was divided into two periods. However, not all regulations were enforced, and as fishers discovered new fishing grounds, fishing effort increased with a completely irregular pattern, resulting in the systematic depletion of several fishing grounds. At the end of 2012 a large unexploited stock was discovered north of the Yucatan Peninsula. This stock was opened for fishing in April 2013, which gave sufficient time to study the fishery and provide elements to improve its management, which are described in the following sections.

### Geostatistical stock assessment

Factors affecting the abundance of *I*. *badionotus* offshore of the Yucatan Peninsula in 2013 were analyzed following two steps: i) geostatistical stock assessment; and ii) bioeconomic modeling. The geostatistical analysis is a continuation of the work presented by Hernández et al. [33], who documented the patchy distribution of *I*. *badionotus* by means of ordinary kriging spatial interpolation. The geostatistical assessment consisted of calculating the abundance of sea cucumber based on its patchy distribution in two different periods. Unlike conventional stock assessment methods addressing population dynamics, geostatistics only estimates one stock’s spatial distribution per period [34]. In this paper, a portion of the *I*. *badionotus* stock off the coast of the state of Yucatan, Mexico was assessed in March and August 2013. These months were deliberately selected since this previously unexploited population was opened to fishing in April 2013. Stock assessments were conducted the month before opening season and again five months later to quantify the fishing impact.

The basic definition of a patchy distribution is a relatively discrete area of a particular spatial pattern with no constraints on its size, internal homogeneity, or discreteness [35]; however, for the purpose of this study patches were delineated following a very specific criterion: the isoline with the minimum value that permitted a closed polygon to be drawn. In geostatistics, a patch can be considered a set of contiguous cells with a common attribute value or classification, so that its area is determined by the number of cells in the clump and the grid resolution. Its perimeter is the external boundary of cells with the same attribute [36]. Once a patch was defined, sea cucumber abundance within it was calculated based on the relationship between animal density, patch area and abundance. Density was defined as abundance divided by the area sampled; therefore, abundance was calculated as density multiplied by area.

Ordinary kriging (OK) models were generated using the TNTmips Basic software package [37]. Density was therefore presented as a continuous field (raster image) in which every pixel was assigned a digital value representing its density. In this paper, the raster image pixels represented 50 m^2^, therefore each pixel’s abundance was calculated by multiplying its size (50 m^2^) by its density value. The total abundance of each patch was therefore obtained as the sum of the abundance values for all the pixels contained within the polygon enclosing the patch.

Subsequent bioeconomic analysis required the creation of areas with different density levels within each patch. A density level corresponded to the surface between two contiguous isopleths in the OK model maps. Each patch encompassed several density levels, the number of which was determined by the number of isopleths within the patch (intervals were pre-set at 0.05 individuals m^-2^, *e*.*g*. 0.05, 0.10… 0.45 individuals m^-2^).

To calculate biomass, the length (cm) and weight (g) of at least ten sea cucumbers collected along each transect were recorded [33]. An average individual weight was inferred from these measurements, and was calculated for each density level by multiplying its abundance (*N*_*i*_) by this average weight (*w*).

### Bioeconomic model

A deterministic and dynamic bioeconomic model was developed to analyze the sea cucumber fishery in the short term [23]. The model included three components or subsystems: biological, technological, and economic. These subsystems interacted to determine the behavior of the whole system over time. The biological component simulated the structure of the patch distribution. The elements of this component were abundance, density, and natural constant mortality. The initial abundance (*N*_0_) was defined as the number of individuals calculated using the corresponding OK model (1 April 2013). It was assumed that the patch was a closed unit, with no migration or recruitment. The patch was divided into eight sub-areas with different average density levels: 0.68, 0.125, 0.175, 0.225, 0.275, 0.325, 0.375, and 0.425 individuals m^-2^. The model used a depletion function (Eq 1) [38] to calculate the trend of abundance, driven by natural and/or fishing mortality, with no changes in the size and shape of sub-areas.

$$N_{i,t}=(N_{i,t-1}-Y_{i,t-1})e^{-m}$$ (1)

where *N*_*i*,*t*_ is the number of individuals at the density level *i* at time *t*, *Y*_*i*,*t*-1_ is the number of individuals removed from the population due to fishing at the density level *i* at time *t*-1, and *m* is the natural mortality. Biomass was calculated by multiplying abundance by the average weight. Density was obtained by dividing abundance by area size.

The technological component was modeled with two main elements: the number of fishing boats per day (fishing effort) and the catchability coefficient, which is the fraction of the sea cucumber population that can be harvested by one unit of effort (fishing trip). After the model was parameterized, the initial number of boats was calculated according to the observed abundance during the two time periods (See Appendix 1). The catch in any sub-area and during time *t* (*Y*_*i*,*t*_) was obtained by multiplying the fishing mortality, i.e. the effort (*f*_*i*,*t*_), by the catchability coefficient (*q*_*i*,*t*_), by the population abundance (*N*_*i*,*t*_) (Eq 2) [39].

$$Y_{i,t}=q_{i,t}f_{i,t}N_{i,t}$$ (2)

The spatial dynamics of fishing effort (in number of fishing trips) was modeled using the Vernon-Smith function [39], which allocates effort spatially according to the income that is obtained during the previous time period from fishing in different areas (Appendix 1). After comparing the incomes obtained in the different fishing areas, the model distributed the number of boats in the areas in proportion to the incomes, as described by Seijo and Caddy [40]; when the income fell to zero in any area, the function stopped allocating fishing effort to it. Catchability was density-dependent, i.e. it was not constant but rather gradually declined as density decreased in each area. This catchability function was suitable and did not vary because sea cucumbers move slowly; once fishers find a bank, they harvest it until it is no longer profitable. Parameterization of the catchability function is described in Appendix 1.

The third component was the economics element. This component was determined by the quasi-profits obtained per fishing trip in sub-area *i* and time *t* (*π*_*i*,*t*_), multiplying the unit price of the species (e.g. US$3.7 per kilo) by the number of sea cucumbers harvested during the fishing trip and subtracting the variable costs (Eq 3).

$$\pi_{i,t}=f_{i,t}(pq_{i,t}B_{i,t}-c)$$ (3)

where *π*_*i*,*t*_ are quasi-profits, *f*_*i*,*t*_ is the fishing effort, *p* is the unit price, *q*_*i*,*t*_ the catchability coefficient, *B*_*i*,*t*_ is the biomass, and *c* is the unit cost. Unlike profit, quasi-profit only involves variable costs, not fixed costs, and is often used in short term decision making [39]. Average variable costs per trip included the costs of crew meals (on average three fishers), and ice required to preserve fresh sea cucumbers. The cost of fuel was calculated as a function of density. Distance can determine differences in fuel costs; however, in the case of this fishery, when density decreases, the time and distance dedicated to searching for sea cucumbers increase significantly, leading to greater fuel consumption. The price was calculated as a function of total sea cucumber landed and delivered to the buyers. This price was for gutted sea cucumbers, which accounts for 60% of the wet weight. This trend of prices was observed during the open season for fishing, therefore by tracking them over time it was possible to calculate the function parameters.

**Fig 1:**
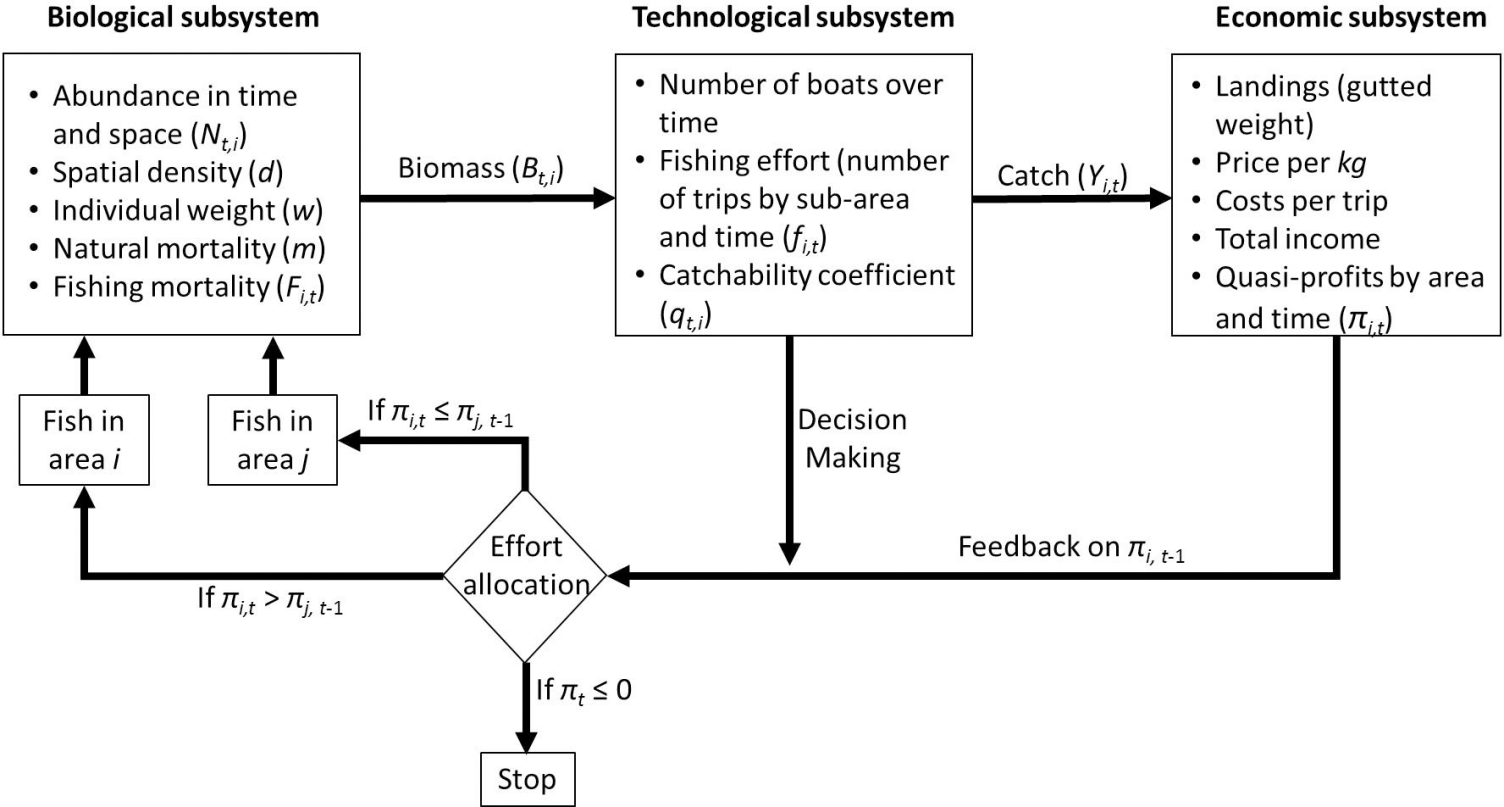
General conceptual spatial-bioeconomic model for the sea cucumber fishery. The three subsystems are connected in such a way that the system functions endogenously.

In summary, the three model subsystems were connected, as the outputs of some components became the inputs of others. These interactions were as follows; i) the biomass available for fishing (biological component) was converted into catch by multiplying total abundance by fishing effort and the catchability coefficient (technological component), ii) the catch was converted into quasi-profits by multiplying it by the price minus operation costs (economic component), iii) quasi-profits determined the intensity of fishing for the next period by area, and iv) the catch was transformed into fishing mortality, which along with natural mortality determined the population abundance for the next period (Fig 1).

## Results

### Geostatistical stock assessment

**Fig 2:**
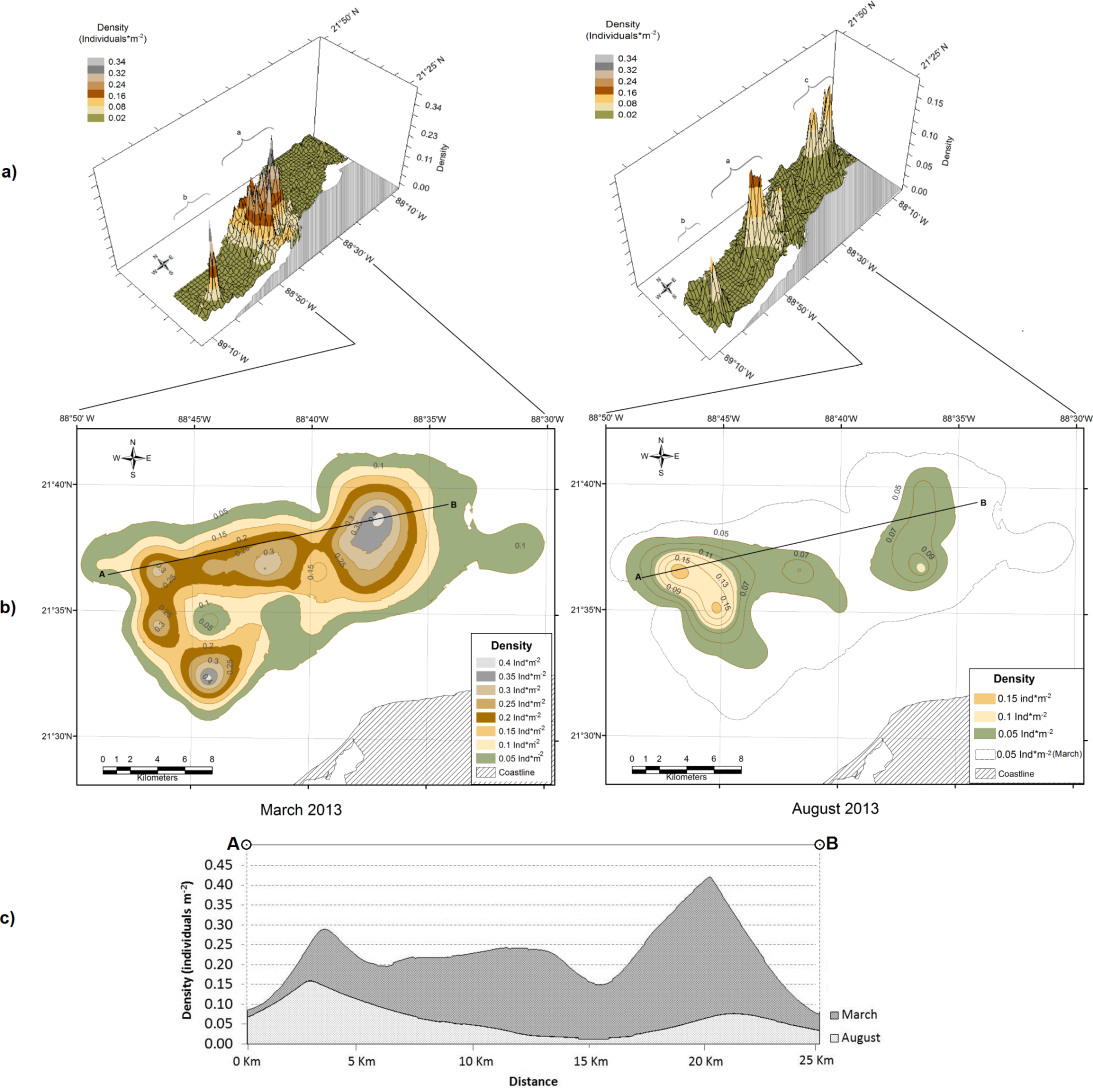
Ordinary kriging models. a) Ordinary kriging models displayed in relief (3D) image format for: March and August 2013. The lower case letters indicate different patches and the colors represent different density ranges; b) Spatial distribution of sea cucumber density in patch *a*, as observed in March and August 2013; c) Profiles of sea cucumber density drawn along the same linear transect (from point A to B) for the two periods of study (March and August 2013).

According to Hernandez et al. [33], an exponential function with ordinary kriging method was used in the spatial model for the March 2013 data, whereas for August a spherical function was applied. The models were displayed in a three-dimensional format (Fig 2A) to illustrate the spatial arrangement of patches and changes that occurred from one period to another. The density isoline with the lowest value to close the OK model patches was 0.05 individuals m^-2^, therefore this value was used to delimit the patches. In March 2013, the most prominent patch (*a*), comprised a vast area with high densities arranged in a complex pattern located in front of Dzilam de Bravo village; the second largest patch (*b*), located toward the west of the area, was significantly smaller and less complex than patch *a*. In August, the model displayed a more complex spatial arrangement as new patches emerged and the density levels of the existing ones decreased. For example, patches *a* and *b* became smaller and fragmented, while new patches emerged toward the east (patch *c*).

In March 2013, the abundance in patch *a* was 51.7 million sea cucumbers, while abundance in patch *b* was 3.7 million, i.e. patch *a* represented 93% of the total abundance; for this reason, the following analyses focuses on patch *a*. This patch presented five peaks in density: two of 0.40 individuals m^-2^, one of 0.35 individuals m^-2^, and two of 0.30 individuals m^-2^. In March 2013, patch *a* covered an area of 221 km^2^. Eight density levels were obtained by applying the criterion of delimiting the patch border at 0.05 individuals m^-2^ and using intervals of the same value (Fig 2B). Five months later, in August, after the fishing season, patch *a* decreased significantly in size and density. By August, patch *a* had split into two smaller areas, one in the east and the other in the west of the original patch (Fig 2B). The total area originally occupied by this patch was reduced by about 63%, and its abundance was reduced by 70%, from 51.7 million to 15.8 million individuals. In order to illustrate the changes in patch *a* due to fishing, the density profiles of along a linear transect were drawn for the two periods (Fig 2C). Further bioeconomic analysis focused on patch *a*.

### Spatial bioeconomic analysis

The bioeconomic model depicted the trajectory followed by the stock during and after the fishing season (from 1 April to 26 August 2013). The performance variables included biomass, density, fishing effort, catch, and quasi-profits, all of which were spatially explicit. Since patch *a*, identified in March 2013, accounted for 93% of the total abundance, the spatial bioeconomic model was focused on the commercial fishing of patch *a*. In order to provide the model with inputs related to the spatial distribution of sea cucumbers, the abundance in this patch was calculated for each density level. The total abundance calculated by the OK model in patch *a* in March 2013 was 51728085 individuals (i.e. 51.7 million), the density level of 0.068 individuals m^-2^ contributed to 14.7% of this value, while the level with 0.225 individuals m^-2^ presented the greatest abundance (20.3%). The density level with 0.425 individuals m^-2^ contributed to less than 1% of the total abundance (Table 1).

**Table 1:** Abundance per density level of patch *a* as calculated by the OK model in March 2013.

Density Level	Initial Density (individuals m^-2^)	Area (m^-2^)	Initial Abundance	^1^Parameter *c*_*i*_ Eq (4)
1	0.425	802792	341187	433106
2	0.375	7073065	2652400	3815919
3	0.325	15031861	4885355	8109689
4	0.275	30024581	8256760	16198261
5	0.225	46682137	10503481	25185013
6	0.175	55090317	9640805	29721226
7	0.125	62916404	7864551	33943400
8	0.068	111522754	7583547	60166526
Total	0.16	329143913	51728085	

^1^*c*_*i*_ = *Area* × *w*, where sea cucumbers average weight is *w* = 0.54 kg

**Fig 3:**
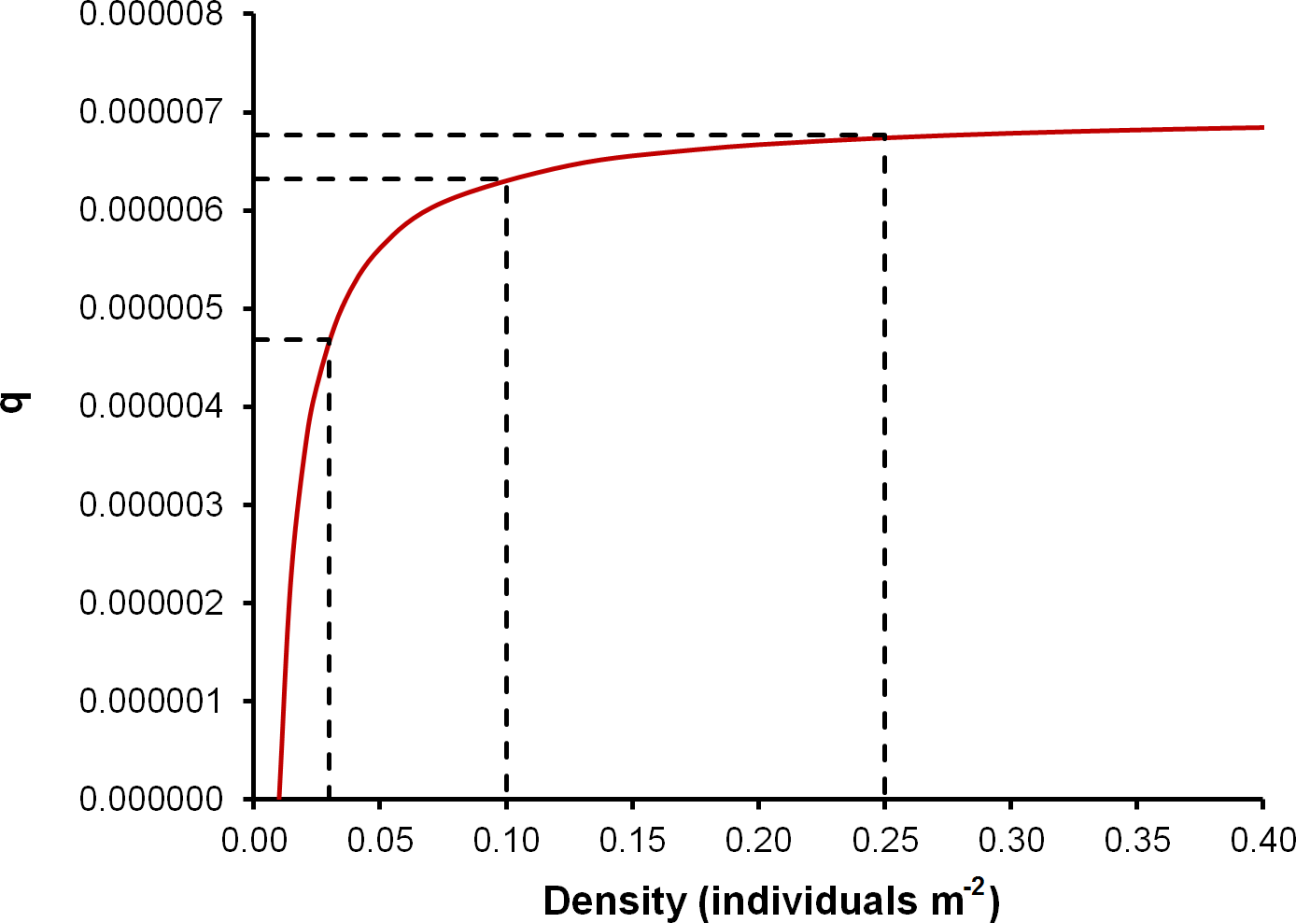
Density-dependent catchability curve of *I*. *badionotus* in patch *a*. Dashed lines show the changes in catchability at three densities: 0.025, 0.10 and 0.25 individuals m^-2^.

These data were used to set the abundance and density values in the initial conditions of the spatial bioeconomic model. The development and parameterization of the density-dependent catchability equation was carried out with field data at both high (*D* > 0.4 individuals m^-2^) and low (*D* = 0.025 individuals m^-2^) densities. Based on these observations the equation (Eq 4) was defined as:

$$q=\frac{aD+b}{c_{i}D}$$ (4)

with three parameters: *a* = 1527.7, *b* = -15.3 and *c*_*i*_, which varies according to the size of the area for a particular density level; *D* is density, which in this case is the independent variable; and *q* is the density dependent catchability coefficient. The values of parameter *c*_*i*_ were also obtained from the OK model (see Table 2). The catchability equation represented a rectangular hyperbola (Fig 3), but since eight different density levels occupied different areas, the *c*_*i*_ parameter prompted the use of eight different equations to simulate the catchability at each density level.

**Table 2:** Parameters used in the model simulation of the fishery in patch *a*.

Parameter	Value	Unit
Natural mortality	0.58	year^-1^
Average weight	539.5	*g*
Initial effort	691	fishing trips day^-1^
Parameter *a* from the catchability function	1527.7	
Parameter *b* from the catchability function	15.3	
Percentage loss from whole to gutted weight	60%	
Annual discount rate	10%	
Slope of the price function	-10.4	
Intercept of the price function	5486	US$
Entry-exit parameter (*ϕ*)	0.035	US$ day^-1^
Parameter *α* of the gas cost function	52.5	
Parameter *β* of the gas cost function	5.2	

Since each level was modeled separately, the model assumed that the area did not vary over time. Furthermore, the catchability equations for each level only differed in terms of the *c*_*i*_ parameter (area of the levels in square meters multiplied by the average weight of the sea cucumbers). Given the definition of catchability as the fraction of stock captured during one fishing trip, its values were specified in terms of the relative abundance for each area. The bioeconomic model reproduced the dynamics of the stock and fishing effort at each level and their interactions among levels, and its parameters are shown in Table 2.

**Fig 4:**
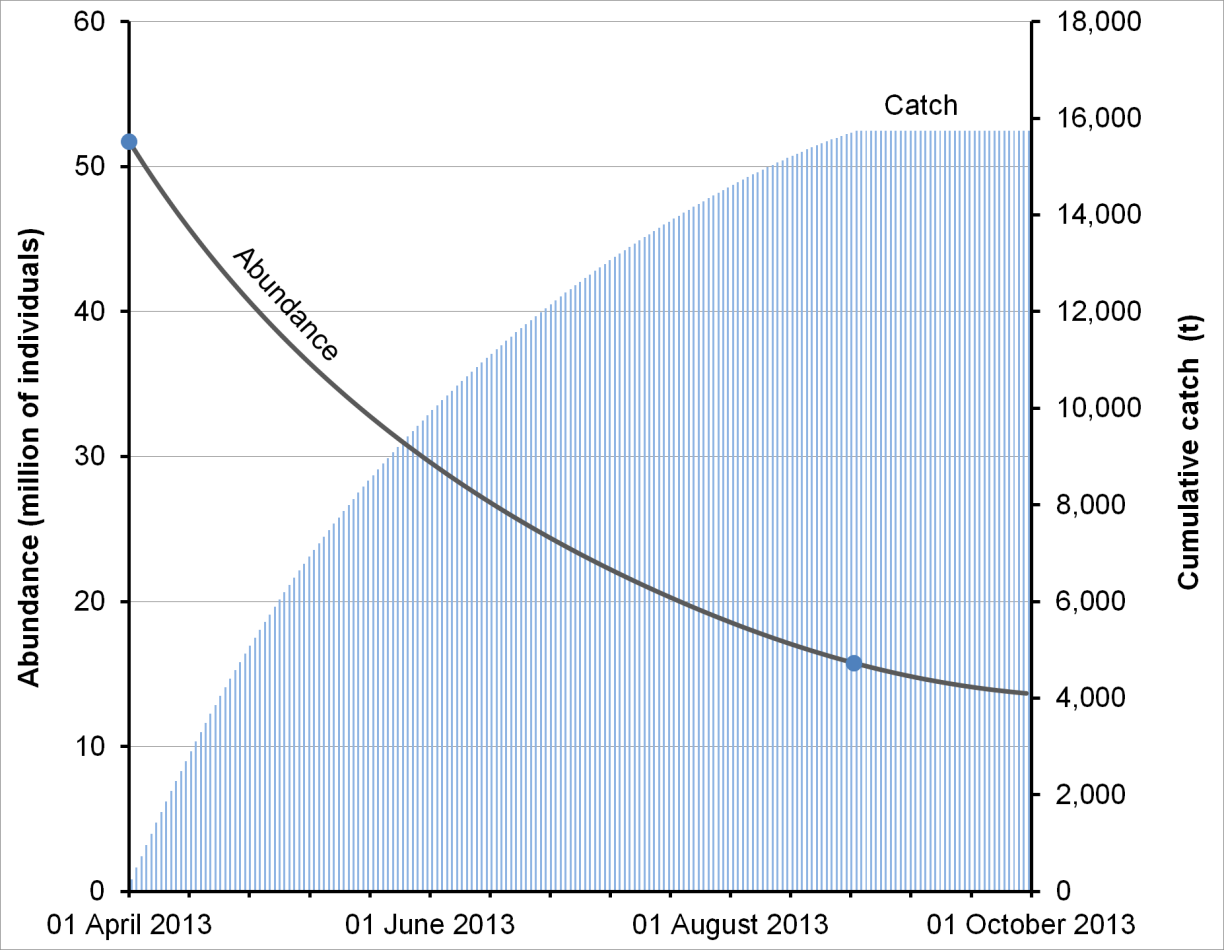
Total abundance of *I*. *badionotus* and cumulative catch trajectories depicted by the bioeconomic simulation model. Total sea cucumber abundance and cumulative catch trajectories reproduced by the bioeconomic simulation model (depletion model) for patch *a*. The blue dots show the observed abundance calculated using the ordinary kriging technique, on 31 March 2013 and five months later (26 August 2013).

The entry-exit parameter of Schmidt’s model was adapted to simulate the decision-making process for the allocation of fishing effort (number of fishing trips per time period) in the short-term (parameter *ϕ*) [39,40]. The model estimated this parameter, as well as the initial fishing effort, by adjusting the trajectory of abundance during the two periods calculated by the OK models, for 31 March and 26 August 2013 (Table 2, Fig 4).

**Fig 5:**
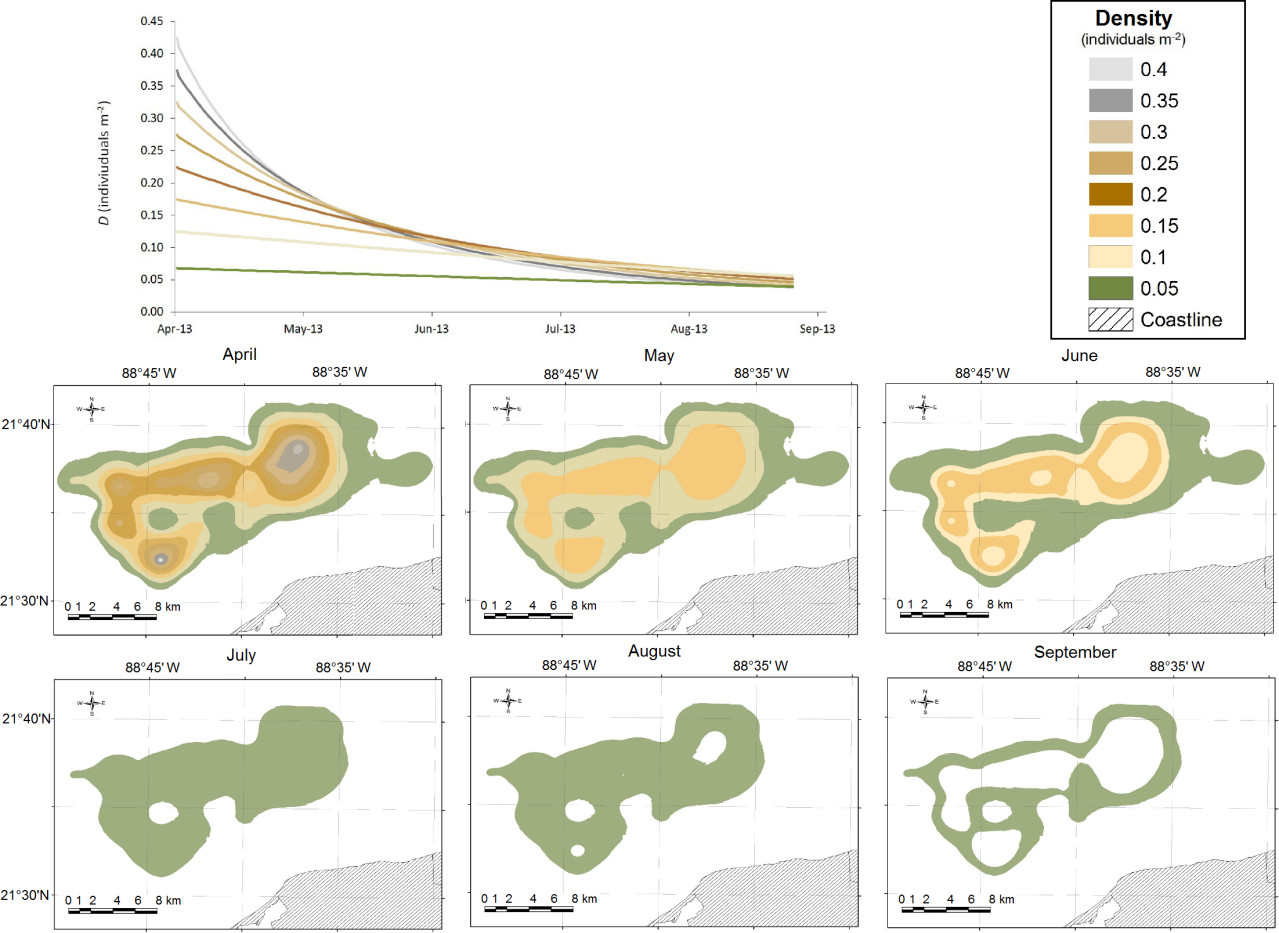
Trajectories of density (*D*) predicted by the dynamic bioeconomic model. Trajectories of density (*D*) predicted by the dynamic bioeconomic model for each of the eight density levels in patch *a*. All densities converge at 0.05 (±0.01) individuals m^-2^ in August. The images at the bottom show the spatial arrangement of density from April to September (on the first day of each month).

The model estimated that from April to August, 2013, 15740 tonnes of sea cucumbers from patch *a* would be extracted by artisanal fishers from different ports of the Yucatan Peninsula. According to the interpolation model there was a 70% reduction in abundance due to fishing during this period. Similarly, the bioeconomic model calculated that in August 2013, the densities of all levels that initially formed the patch would converge in a range between 0.04 and 0.06 individuals m^-2^ (Fig 5). After that time, the bioeconomic model did not show significant changes in biomass as a result of fishing effort reduction.

**Fig 6:**
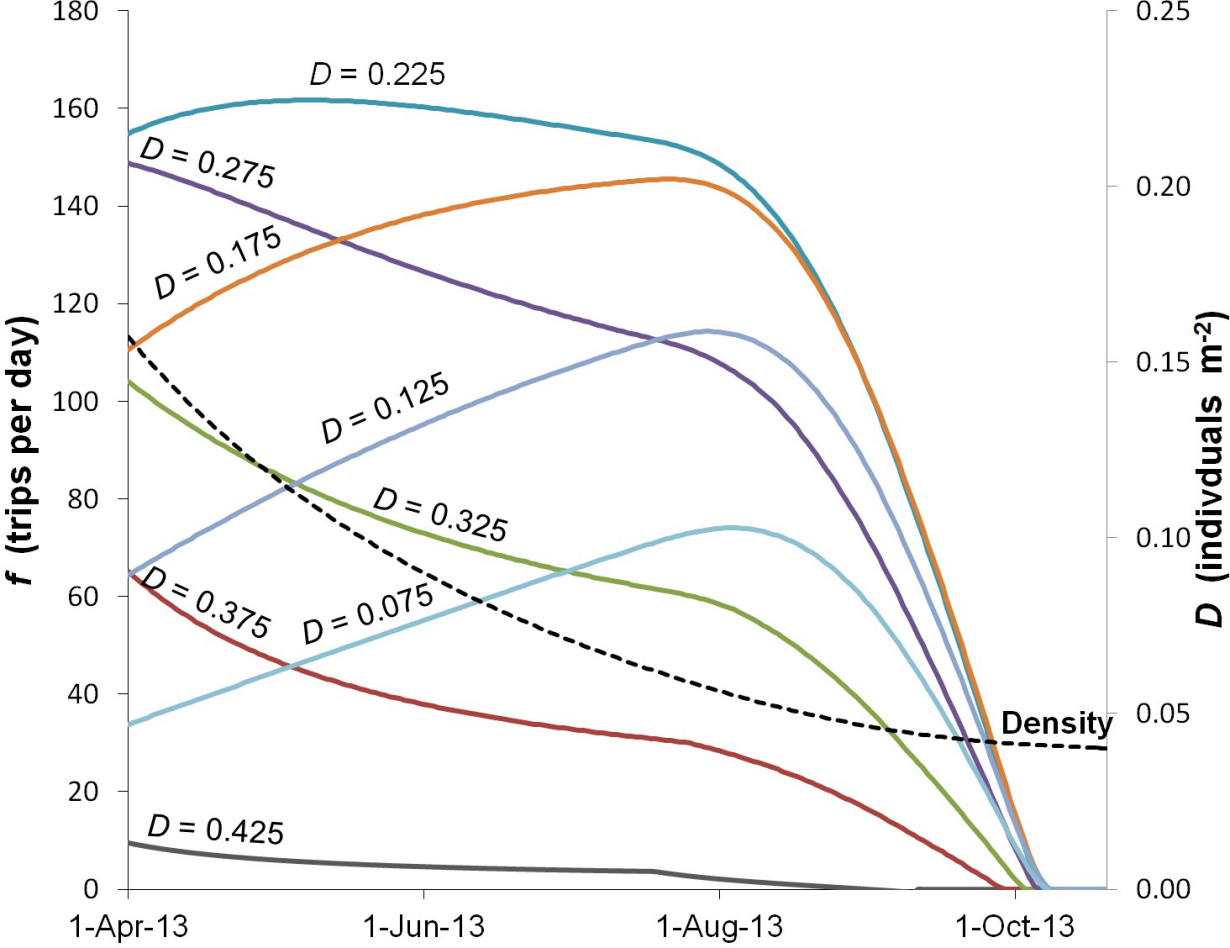
Spatial allocation of fishing effort in patch *a* as portrayed by the bioeconomic model. Each color represents a density level. The trajectory of total density (*D*) fell from 0.16 to 0.04 individuals m^-2^ in six months.

Regarding fishing effort, the bioeconomic model calculated that 691 boats were present at the beginning of the fishing season; these boats were distributed heterogeneously over the different levels of the patch, according to the catch per trip and the costs. As fishing season progressed, the boats moved from less to more profitable areas (Fig 6), although the total effort was kept constant until July (111 days of fishing), after which all boats stopped fishing (during a period of 80 days). It is important to note that the bioeconomic simulation model did not consider the moratorium on the sea cucumber fishery declared by the authorities, after 20 days of fishing. The model showed that it would be unlikely that 350 fishing boats would reduce the stock from 51.7 million to 17.6 million in 20 days. The quasi-profits were the main driver of fishing effort and consequently of stock size, which determined the spatial allocation of effort as well as fishing intensity over time.

In the four levels with the lowest density, the effort increased immediately after fishing started. At a certain point, the effort in all density levels began to decrease and finally ceased. On the other hand, the price tended to increase as the abundance and catch decreased. Initial price was US$2.90 per kg and the final price was US$5.12 per kg (gutted weight).

## Discussion

To our understanding, this is the first time that an adult sea cucumber stock assessment has used the density-to-volume ratio, which was facilitated by the use of geostatistical software. This technique is frequently applied to calculate irregular land volumes [41]. In this sense, the use of this technique to evaluate sea cucumber stocks can be reliable as the sampling design represents the area of distribution of the stock in a relatively short time period (e.g. 10 days) and the organisms exhibit a patchy distribution, given their limited displacements. This technique may not be reliable if the patch distribution becomes fragmented, or if the sampling coverage is insufficient or inadequate. The volume of the patch calculated by the TNTmips software was precise [41] at the spatial scale of the patch distribution, because the models had a resolution of 50 m (pixel size). The precision of the stock assessment obtained by the geostatistics based method depended on the quantity, quality and distribution of sampling data, goodness of fit of the OK model, and spatial resolution.

Although commercial fishing of sea cucumber officially began in the Yucatan Peninsula in 2012, the exploitation of the stock analyzed in this study, which was located off the coast of Dzilam de Bravo village, started after April 2013. For this reason, it has been defined as the initial condition in this study. The OK model for March 2013 estimated that patch *a* would account for 93% of the total abundance of patches present in the study area. The abundance for patch *a* was 51.7 million sea cucumbers, equivalent to 27907 tonnes of biomass (wet weight) in March 2013, and 15.8 million, 8505 tonnes (wet weight) in August 2013, after the impact of fishing; which represented a 70% reduction of the initial stock in five months.

With the total abundance of *I*. *badionotus* calculated for the two periods separated by 148 days, the bioeconomic model reconstructed the trajectory of abundance, biomass, fishing effort, catch and the economic variables of the fishery between the two periods (Fig 4). The bioeconomic model showed that a reduction of 70% in biomass between these two periods would require a total effort of 100371 fishing trips which, divided by the fishing days, gave an average of 678 fishing trips per day. Given that the open season for fishing officially lasted 20 days, the total fishing effort to harvest 15.7 thousand tonnes over that period would be more than 5000 fishing trips per day, which is virtually impossible. As the total allowable catch established by authorities was 2100 tonnes, it is likely that 13.6 thousand tonnes had been harvested illegally. This figure is consistent with the numerous cases of sea cucumber trafficked illegally and filed with the corresponding authorities during the period of the study and later.

The bioeconomic model indicated that fishing effort started at its highest level (691 fishing trips per day), and that this level would be maintained during the following 111 days, after which it would fall gradually. The intensity of fishing effort responded to the quasi-profits; therefore, changes in abundance across space defined the allocation of effort (Fig 6). Fishing effort decreased as quasi-profits tended to zero, and fishing effort completely ceased after 191 days, when the abundance was 13.5 million and density was 0.04 individuals m^-2^.

The ex-vessel price was another factor that exerted a positive influence on fishing effort intensity, with increased prices as the catch decreased, however, this influence was limited by the market forces. The price started at US$2.90 per kg (gutted weight) on day one of the opening fishing season, and increased to US$5.12 per kg on day 148. The fishing effort dynamics in space and time resulted in a sudden reduction and a homogenization of the patch’s abundance (Fig 5).

As the fishing season progressed, quasi-profits fell at a rate similar to that of the decrease in abundance. The reduction in abundance was related to fishing effort intensity, which depended on the profits of the previous day. At high abundance, catchability experienced slight variations as abundance decreased, but it fell drastically at lower densities (*D*<0.07). Various density dependent catchability models have been mentioned in the literature and discussed for sedentary resources, both in theoretical and empirical studies [42,43]. In the present study, the proposed model parameterization for catchability was simple, direct, and based on the relationship between the catch per unit effort and the density by means of a rectangular hyperbola [44–46]. As is the case for other fishing resources with depensatory mechanisms related to species behavior (e.g. Peruvian anchovy) [47], the non-linearity between catchability and abundance may lead to errors in abundance estimation. The relationship between catchability and density may have lead fishers to believe that abundance was still high when in fact it was low. As a result, there was excess fishing effort (beyond the stock capacity), thus it is very easy to drive this stock and other types of sedentary stocks to over-exploitation [22,29,48–50].

According to our results, a significant reduction in the average density of patch *a* from 0.16 individuals m^-2^ on 1 April to 0.05 individuals m^-2^ on 26 August 2013 occurred over 148 days. A similar reduction was documented by Hasan and El-Rady [51] in the Red Sea for the density of black teatfish (*Holothuria nobilis*, Holothuriidae); 0.167 individuals m^-2^ in 1995 and 0.007 individuals m^-2^ in 2002. These authors reported that the species disappeared altogether in both 2003 and 2006. The density of sandfish (*Holothuria scabra*, Holothuriidae) was also high (0.194 individuals m^-2^) in 1995, but decreased to 0.011 individuals m^-2^ in 2002 before completely disappearing in 2003 and 2006 [51].

This study confirms previous findings [7,29] that sea cucumbers are highly vulnerable to overfishing, particularly in small-scale fisheries, due to the high quasi-profits that fishers may obtain when targeting virgin stocks. The bioeconomic model presented in this study suggests that the range of yield per trip at the beginning of the fishing season (April 2013) was from 157 kg to 634 kg, depending on the density of the fishing ground. These catches produced quasi-profits of US$526 per trip (minimum of US$101, and maximum of US$996); these values are extremely high in comparison to the fishers’ opportunity costs (average US$85) obtained by targeting alternative resources in the region, such as finfish. In theory, fishers will no longer capture sea cucumbers as their quasi-profits become equal to or lower than the opportunity cost [22,43]; instead, they will target another species. Despite the fact that the bioeconomic model indicated that the fishery would reach a minimum density of 0.04 individuals m^2^, it is likely that the stock continued to fall, as has occurred in other regions [7,51]. This decline in stock may be the result of illegal fishing as poachers obtained higher prices on the “black market”.

Given that a reduction of 70% of the biomass in five months is a serious threat to the stock, the bioeconomic model shed some light on alternative regulations. For example, a cap on daily fishing effort would effectively reduce fishing mortality and catches, while the product could reach higher prices. In this regard, an alternative scenario would be to set a maximum effort between 31 and 287 fishing trips per day; these are the numbers required to achieve annual catches of 5% and 50% of the initial biomass, respectively, assuming the virgin biomass of the patch is 27907 tonnes. According to Uthicke et al. [3] the precautionary approach recommends annual catches of up to 5% of the virgin biomass, which in the present study represents 1384 tonnes.

The bioeconomic model demonstrated that maintaining a maximum effort of 31 fishing trips per day would have produced a net present value of US$3.9 million, 17% of the *status quo* (Table 3). Otherwise, maintaining a maximum effort of 287 fishing trips per day would have produced a net present value of US$20.8 million, which is 90% of the *status quo*. In this last scenario, the price would fluctuate between US$4.41 and US$5.10 per kg. Although there were 691 boats participating in the fishery, limiting effort to 287 fishing trips per day in a rotation system with a random selection process, would tackle the “race for fish” and the inequity problem. This would allow all users to access the resource without detriment to it, while providing benefits to all participants [8]. By controlling landings through the limitation of daily fishing effort, fishermen could have more influence on prices, avoid sudden drops in stock and obtain higher profits. In such a situation, the expected buyers’ strategy is to marginally increase the price as an incentive to increase the fishing effort. As a result, landings increase in the short term, with a significant increase in profits generated by a greater amount of sea cucumber exported to Asia. For this reason, community agreements become fundamental to achieving social goals.

**Table 3:** Comparative analysis between the current situation (status quo) and alternative strategies to limit the maximum fishing effort per day to yield a catch of 5% (31 fishing trips) and 50% (287 fishing trips) of the initial biomass, with different performance variables of the bioeconomic model.

		Effort limit (fishing trips day^-1^)	
	*Status quo*	31 fishing trips	287 fishing trips
Total fishing trips	100371	4588	42476
Remaining Abundance (millions)	15.8	38.5	25.9
Density (individuals m^-2^)	0.05	0.12	0.08
Cumulative Catch (t)	15740	1384	9274
Cumulative NPV (US$ million)	23.0	3.9	20.8
Price (US$ kg^-1^)	5.12	5.40	5.10
Avg. income per trip (US$ trip^-1^)	230	849	490
Total Avg. income per boat (US$ boat^-1^)	33355*	5636*	30092*

* This calculation is based on the assumption that the 691 boats participate with an equitable distribution of the benefits

NPV = Net present value

This regulation, together with the existing ones such as season closures, minimum legal size, prohibition of evisceration on board, agreements to define fishing days during the week, and catch limit per trip, will not be sufficient to stop excess fishing effort if fishing communities do not participate in decision-making processes to take responsibility for the sustainable use of the resource [52,53]. The race for sea cucumbers and illegal fishing was motivated by a reduced number of licenses and the high number of fishers who already existed in the region. Other factors that motivated illegal fishing were: the high price that sea cucumbers reached, buyers strategies, and the lack of scientific knowledge about the resource. In practice, fishing occurred under open access as fishers thought that no one was going to abide by the regulations.

The scientific knowledge about the resource is a key element to consider when resource regulations are being established and legitimized. Moving towards co-management would require the scale of fishing operations to be adjusted, such as the number of licenses issued. Greater equity promoted by an orderly access of the majority of fishers to the resource could contribute to reducing pressure and enforcement costs. This would require raising awareness among stakeholders through communication campaigns and public hearings. It is important that stakeholders understand that this type of resource cannot support a high fishing effort given its biological characteristics and the time it requires to grow and reproduce. More fishers convinced of the importance of implementing better management practices would have greater social influence and exercise a moral obligation that would define users’ behavior and could contribute to better compliance [54]. Additional aspects to be integrated include: the allocation of authority to fishers, voluntary participation of fishers in enforcement, improving scientific knowledge, and incorporation of communities’ traditions and idiosyncrasies [50,53,55]. The challenge for managers is to meet these criteria with the aim of reducing the over-exploitation threat, the losses produced by poor processing and wastes, social conflicts, poaching, and social decomposition.

## Conclusions

The reduction of 70% of the total abundance of sea cucumber in patch *a*, in 148 days (from March to August 2013) is attributable to the fishing impact. The spatial distribution pattern of the remaining stock was affected; the new spatial distribution pattern was the result of the original distribution (natural) and spatial allocation of effort that focused on fishing areas with higher densities, leaving an average density of 0.05 individuals m^-2^. Since these organisms have a relatively low mobility rate, density experienced a drastic reduction. If fishing were to continue, it is likely that the patch would disappear in the short term.

The main factors that could lead to *I*. *badionotus* depletion are: a) the spatial pattern displayed, in relation to their adaptations, b) excess fishing capacity, c) high quasi-profits per fishing trip in the short term, d) the density dependent catchability coefficient, and e) the heavy density dependence of reproductive success. These features make this and other species of sea cucumber highly vulnerable to overfishing.

Because the opportunity cost of Mexican fishers is very low compared to the high income that they can obtain from catches per unit effort in the short term, and their high discount rate, they preferred to maximize their short-term quasi-profits, regardless of the losses that this represents in the long run. In this sense, profit maximization does not consider the ability to renew the stock in the long term; thus, under an open access regime, this type of fishery tends to systematically deplete the different patches as they appear, which could result in the total collapse of the resource in a few years. In short, the decline in most fisheries of sea cucumbers worldwide can only be reversed by a strict control of fishing mortality, which maintains populations while taking care of densities to ensure successful reproduction. Therefore, fishermen's participation in the decision-making processes is crucial for the sustainable use of the resource. It is essential to understand the ability of species to recover from the impacts of fishing; for this reason it is important to continue with research on density-dependent reproductive processes, on the Allee effect and on the stock-recruitment relationships.

## Supporting information

S1 Appendix Formulation of the spatial dynamic bioeconomic model. (DOCX)
