# Supplementary material for: Bioeconomic modeling for a small-scale sea cucumber fishery in Yucatan, Mexico
Source: PLoS One. 2018 Jan 9;13(1):e0190857. doi: 10.1371/journal.pone.0190857 (PMC5760041; doi:10.1371/journal.pone.0190857)
Supplement: S1 Appendix — (DOCX) [file pone.0190857.s001.docx]

**Appendix 1**

**Formulation of the spatial dynamic bioeconomic model**

The population dynamic of the Yucatan *I. badionotus* fishery was analyzed by treating the patch as a stock unit. The stock as quantified in patches was simulated over a six-month period with a simple depletion dynamic model:

*N_i,t_* = (*N_i,t_*_-1_ – *Y_i,t_*_-1_) *e*^-^*^m^* (1)

Where *N_i,t_* is the number of individuals at the density level *i* in time *t*, *Y_i,t_*_-1_ is the number of individuals removed from the population due to fishing at the density level *i* in time *t*-1, and *m* is the natural mortality [38]. The model assumed a closed population (i.e. the patch), and no recruitment, all reasonable assumptions given the short simulation period (six months) and this species’ normal lifespan (10+ years) [32]. The six-month period spanned from 1 April 2013 to 26 January 2014, but in all cases fishing effort collapsed before completing the full term. Natural mortality was calculated using the method proposed by Rithker and Efanov [56].

Initial abundance was taken from that calculated with the OK model for 1 March, and, based on field data, patch abundance was known up to 26 August. Previous studies have used depletion models to calculate a resource’s initial population and catchability coefficient (*e*.*g*. [57,58]). The fact that initial population and one population level during the period were known in the present study allowed use of the depletion model to predict catch trajectory and other bioeconomic variables. Model was built in a spreadsheet (Excel®) and parameterization was done by calculating initial fishing effort expressed in number of fishing trips day^-1^, given initial and final abundance. The problem consisted in finding the initial effort that should exist to drive the stock from initial and final abundance, given catchability, price and costs functions independently estimated. The model included an algorithm that defined the spatial allocation of fishing effort according to previous quasi- profits. Because this is a nonlinear model, and in order to find the solution, we used an iterative method (goal-seek function of Excel®), which gave the exact solution to the problem. However, and because the fishing trips are indivisible, we rounded the solution to the entire number of fishing boats working in day zero of the simulation (1 March).

Except for the initial conditions, the model was fully endogenous, meaning that once initialized the biological, technological and economic subsystems interacted within each period to produce the conditions for the following period until the simulation terminated.

The spatial bioeconomic model divided the patch into sub-areas corresponding to the density levels calculated by the OK model for March. In contrast to the OK model, the spatial bioeconomic model had the limitation that it assumed uniform density throughout each band, using the midpoint of the OK model density range as the uniform density of a band. For example, for a density band with a range of 0.1 to 0.15 individuals m^-2^ the constant density value was 0.125 individuals m^-2^.

Initial conditions for the spatial bioeconomic model included abundance in each density band, a process analogous to that proposed by Anderson and Seijo [43] for a spatially-distributed population of a single stock. Model time unit was one day. As time progressed in the model, abundance in each density level over time was calculated by subtracting the number of sea cucumbers harvested the previous day minus deaths by natural mortality, using the equation

*N_i,t_ =* (*D_i,t_ A_i_ – Y_i,t-1_*) *e*^-^*^m^* (2)

Where *N_i,t_* is the number of individuals in band *i* at time *t*; *D_i,t_* is density in band *i* at time *t*; *A_i_* is the area of band *i*; *Y_i,t-1_* is the catch in band *i* at time *t-1*; and *m* is the natural mortality. Biomass in each density level was calculated by multiplying the number of individuals by average individual weight. For initial conditions, the density in each level was calculated from the OK model.

Catch was calculated with the basic Schaefer equation [22], but with an independent estimate of catchability (*q*). Several authors have noted problems when using a biased catchability coefficient since it changes in response to abundance [38]. To address this issue, a simple procedure to calculate the density-dependent catchability coefficient was used. Based on the Schaefer model, effort (*f*) was fixed at one unit (*f* = 1 fishing trip day^-1^); thus, at one site with known biomass and density, catch was:

*Y_i_* = *q_i_ B_i_* (3)

The subscript *i* indicates any site with a specific density. To generate a proxy for the density-dependent catchability function, logbooks were analyzed at the beginning of the fishing season and compared to maximum observed density (*D_iMax_*). Calculations were also done of minimum density (*D_iMin_*), that is, the density at which fishers stopped catching sea cucumber due to an inability to cover trip variable costs. Using these two data extremes, *q_i_* could be extracted from (3):

$q_{i}=\frac{Y_{i}}{B_{i}}$ (4)

Previous observations in areas near the virgin patches studied here indicated that divers (fishers) could not cover trip variable costs at sites with densities ≤0.02 individuals m^-2^. This density was assumed to be the catch limit (*D_i,Min_*,), and a straight line extrapolated from the point of maximum density *D_i,Max_* to *D_i,Min_*. The regression equation between these two points, maintaining effort equal to one trip per day at sea, was:

$Y_{i}=aD_{i}-b$ (5)

Where *D_i_* is the density at site *i*; *a* is the slope; and *b* the intercept.

Under the assumption that a fishing area with a known density did not change its size, biomass in that area changed in direct proportion to density as the resource was harvested:

*B_i_* = *c_i_D_i_*  (6)

Where *c_i_* is specific to an area with a particular density level, and corresponds to the slope of this relationship (note that the origin is zero). It is important to mention that *c_i_* was equal to the product of the average weight of sea cucumbers multiplied by the total area of each density level. Substituting (5) and (6) in (4) results in:

$q_{i}={a_{i}}^{'}- \frac{b}{c_{i}D_{i}}$ (7)

Where:

$${a_{i}}^{'}=\frac{a}{c_{i}}$$

Equation (7) corresponds to a rectangular hyperbolic function with *a_i_′* as the asymptotic term, *i.e.*, maximum catchability per unit effort, regardless of density. *c_i_* is the slope for site *i*, and *b* is the same intercept as in Equation (5). Different authors have explored hyperbolic functions to model the predator-prey relationship, treating fishers as predators and target species as prey [59]. Of the three types of predator-prey relationships reviewed, the hyperbolic function applied here is a Type II function [46,60].

The bioeconomic model’s spatial aspect is provided by the fact that it is analyzed by density levels. The biological, fishing and economic variables are projected over time through a series of difference equations. Initial model conditions were based on the patch’s virgin stock as determined by the OK model for March 2013. Density over time was the number of individuals in a density level divided by that level’s total area (constant *c_i_* in Equations 6 and 7). Because catchability is density-dependent, it changed over time in response to density, and was derived from Equation (7):

$q_{i,t+1}={a_{i}}^{'}- \frac{b}{c_{i}D_{i,t+1}}$ (8)

Catch was then calculated with the Schaeffer equation by site (density band) and time:

$Y_{i,t}=q_{i,t}f_{i,t}+B_{i,t}$ (9)

Where *f_i,t_* is effort at site *i* and time *t*. Effort was distributed in space based on the proportion of profits earned at each band density versus total profits [39]; this same procedure was operated dynamically. The dynamics of total effort (number of boats per day) was simulated with the Vernon-Smith equation [43]:

$\frac{dE}{dt}=\phi\left[ E(pqB-c) \right]$ (10)

Where *ϕ* > 0 is the entry-exit parameter; *p* is price; *q* is the catchability coefficient; *B* is biomass; and *c* is cost. The entry and exit parameter (*ϕ*) was calculated by approximation, making *f* = 0 when quasi-profits were zero. Quasi-profits per boat at site *i* and time *t* (*Π_i, t_*) were calculated by subtracting total variable costs (*TVC_i, t_*) from total revenues (*TR_i, t_*). Total revenues were obtained by multiplying catch (*Y_t_*) by the ex-vessel price, which was monitored throughout the season and varied in response to production. The model simulated changes in sea cucumber price as a linear function the parameters for which were obtained from a regression of price on landings. Price was lowest when landings were highest at the beginning of the fishing season, and became higher as landings decreased, resulting in a demand curve. Fishers had to deliver gutted sea cucumbers, so the price corresponded to gutted weight. Total capture (whole weight) was therefore multiplied by 0.6 to convert the 40% loss to whole weight from gutting into gutted weight. Variable costs per trip (day at sea) included the costs of gas, ice and meals for fishers, and were averaged from a survey of 883 fishing trips. A cost density-distance transfer function was applied to calculate gas cost per trip, a variable linked to trip time which in turn depended on the density of harvested sea cucumbers:

$c_{gas}=\alpha+\frac{\beta}{D}$ (11)

Where *c_gas_* is the gas cost per trip, which is inversely proportional to sea cucumber density (*D*); *α* and *β* are constants.
